# Extending basic principles of measurement models to the design and validation of Patient Reported Outcomes

**DOI:** 10.1186/1477-7525-4-65

**Published:** 2006-09-22

**Authors:** Mark J Atkinson, Richard D Lennox

**Affiliations:** 1 Worldwide Health Outcomes Research, La Jolla Laboratories, Pfizer Inc., San Diego, CA 92121, US; 2 Health Services Research Center, USCD School of Medicine, La Jolla, CA 92093, US; 3 Psychometric Technologies, Inc., 402 Millstone Drive, Suite A, Hillsborough, NC 27278, US

## Abstract

A recently published article by the Scientific Advisory Committee of the Medical Outcomes Trust presents guidelines for selecting and evaluating health status and health-related quality of life measures used in health outcomes research. In their article, they propose a number of validation and performance criteria with which to evaluate such self-report measures. We provide an alternate, yet complementary, perspective by extending the types of measurement models which are available to the instrument designer. During psychometric development or selection of a Patient Reported Outcome measure it is necessary to determine which, of the five types of measurement models, the measure is based on; 1) a Multiple Effect Indicator model, 2) a Multiple Cause Indicator model, 3) a Single Item Effect Indicator model, 4) a Single Item Cause Indicator model, or 5) a Mixed Multiple Indicator model. Specification of the measurement model has a major influence on decisions about item and scale design, the appropriate application of statistical validation methods, and the suitability of the resulting measure for a particular use in clinical and population-based outcomes research activities.

## Background

Over the past two decades, health outcomes researchers have tried to present convincing evidence to regulatory agencies and healthcare planners that Patient Reported Outcomes (PROs) provide a benefit beyond the assessment of clinical outcomes alone. This persistent belief in the added value of patients' ratings of illness and treatment has resulted in a continual refinement of PROs measures for use in clinical settings. Although conceptually and philosophically appealing, widespread acceptance of PROs has generally proved to be a challenge in regulatory and clinical environments, where a high value is placed on biomedical outcomes and where skepticism persists about the meaningfulness of such concepts such as quality of life, treatment satisfaction, and symptom distress. Contributing to such reluctance, some existing PRO measures have been openly criticized in the research literature as being inadequately conceptualized, lacking psychometric rigor, and based on inconsistently applied psychometric methods [1,2]. Such criticisms further undermine the credibility of PRO measures and tend to sideline their application in mainstream clinical research [3].

Various stakeholder groups have attempted to address the situation by providing PRO development guidelines with which to evaluate and construct PRO measures. An influential example of such guidance was recently published by the Scientific Advisory Committee (SAC) of the Medical Outcomes Trust [4]. In this guidance the SAC states that PROs should be evaluated on the following seven dimensions; 1) the use of pre-specified conceptual and measurement models; 2) the strength of empirical support for the reliability and validity of the scale(s); 3) the responsiveness of PRO to clinical change; 4) the method(s) for interpreting scores; 5) the level of respondent and administrative burden; 6) the equivalence of alternative forms of administration; and 7) the rigor with which translations are adapted for use in specific cultural contexts. This list appears to be comprehensive but there is at least one aspect of their recommendations, namely the prior specification of conceptual and measurement models, that warrants further consideration.

The SAC rightly advises that measurement models be used to describe the logical and empirical basis for item combination, yet the guidance focuses almost solely on the use of factor and item-response analyses as appropriate methods for instrument design and construct validation. An inadequate coverage of important alternatives to classical factorial models; such as the use of linear combinations of independent items, single item indicators, or other scalar metrics, seems to imply that there is only one suitable type of measurement model to structurally model PRO scales [5]. While it is true that many outcomes researchers do not view these other measurement models to be as structurally sound as classical psychometric methods, the use of alternate models has been shown to result in measures which are better suited to the purposes of diagnostic differentiation, symptom severity rating, epidemiological investigation, and clinical decision-making [6-13]. If this point were better understood and applied within the field of Health Outcomes Research, PRO measures might well find greater acceptance among the broader medical research and clinical practice communities.

The purpose of this commentary is to review basic conceptual and statistical principles associated with a variety of different types of measurement models. In the first section (Part I), we use very basic structural equation diagrams to depict the statistical features associated with four prototypical forms of models. The concept of a "family of measurement models" is presented to delineate important distinctions between the cause and effect relationships among PRO items, scales, and health-related phenomena of interest. In Part II, we elaborate on how the measurement assumptions associated with the various models influence both their psychometric characteristics and validation requirements. It is our hope that improved structural specification of PRO measures will result in greater measurement precision and promote a deeper appreciation of patient-reported measurements methodologies being used in other areas of clinical and epidemiological research.

## Part I: Latent constructs and measurement models

In the 3^rd ^edition of their classic reference, *Psychometric Theory*, Nunnally and Bernstein (1994) suggest that the use of informant-based measures is necessarily focused on measuring latent constructs rather than on observable phenomena [14]. They point out that many biological, psychological and social states are, by nature, unobservable and must be inferred (e.g., physical pain, emotional well-being, satisfaction etc.). As a result, investigators rely on observable indications to infer individuals' standing on these unobservable latent constructs. The term 'latent' is used to emphasize that any set of measured observations, no matter how precise and elegant, is only an indirect approximation of an unobservable construct, and that all relevant observations are necessarily one step removed from the construct they are designed to measure.

Due to the indirect and inferential nature of PRO constructs, the validity of a measure is never simply demonstrated by reliable observation but must also shown to exist through confirmation of a measures' theoretical relationship to other established constructs or objective criteria. Two founders of psychometric theory, Crohbach and Meehl [15] coined the term 'nomologic net' to describe the theory-building effects of construct validation activities; activities which act to continually expand a theoretical network of inter-related concepts [16,17]. For example, evidence for a construct of social function could include demonstration of its association with established measures of both physical impairment and social support. Similarly, the associations observed between patients' responses to different items may provide evidence for the existence of an underlying and organizing construct. Specification of the relationships between items and the latent construct(s) define measurement models and these models are in turn used to help demonstrate the structural validity of new PRO scales.

Thus two characteristics are hallmarks of well designed measures, valid observational (i.e., item) content and confirmation of a structural relationship between items and the measurement construct. The first, content validity, is demonstrated by qualitative and quantitative evidence that items assess content that patients perceive is relevant to the construct of interest. In turn, structural validation efforts demonstrate how patients' ratings on these items are to be statistically related to each other so as to estimate the underlying construct. Modeling of the relationships between items and measurement constructs is most clearly depicted using structural equation notation.

**Figure 1:**
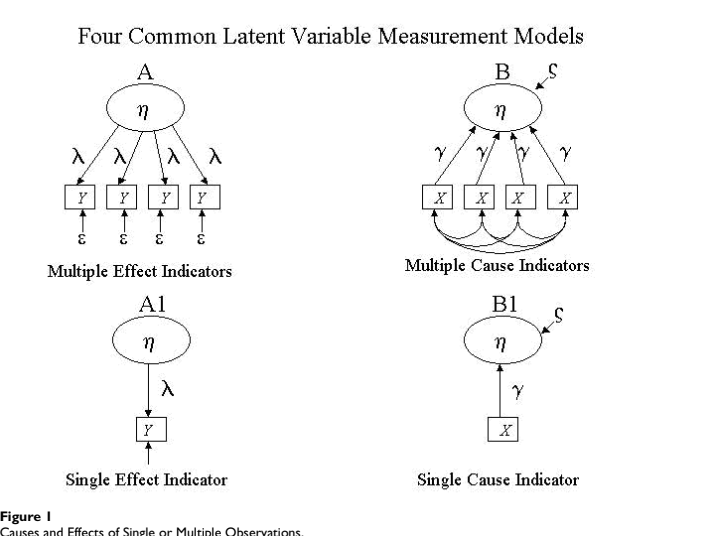
Causes and Effects of Single or Multiple Observations.

Although complex forms of latent construct models are used in the social science and psychological literatures, so as to simplify discussion, the four basic elements common to all measurement models are presented in Figure 1. We begin by describing these four *families *of measurement models and provide a practical example of a PRO scale based on each.

### Multiple Effect Indicator models

Starting with the top left quadrant of Figure 1 with Model A, the Multiple Effect Indicator (MEI) model represents what most people commonly refer to as the latent construct or factorial model. In this model, the presumed relationship between the latent construct and the measured items is explicit, that is the factor loadings for each item (λ) represent the extent to which the variance in each item is explained by a common factor, a factor which is defined by the common variance across the entire set of items. The explicit relationship between the latent construct and the covariance between observed items can be expressed as in equation [1]:

*Y*_i _= λ_I1_η_1 _+ ε_1 _    [1]

Where *Y*_i _represents the covariance in the measured items, η_1 _represents the underlying latent construct, which indexes the statistical intersection of all indicators, λ_I1 _indexes the statistical relationship between the latent construct and the measured items, and the ε_1 _represents the random measurement error in the i^th ^measured item.

**Figure 2:**
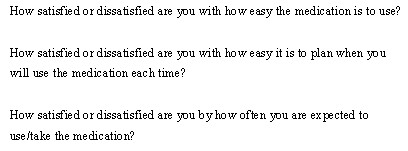
Items comprising the TSQM Convenience Scale.

The Convenience scale of the Treatment Satisfaction Questionnaire for Medication (TSQM v. 1.4) is an example of a PRO scale that is based on an MEI model [18]. Shown below, three items comprise the Convenience scale, and are intended to measure patients' satisfaction or dissatisfaction with the convenience-inconvenience of medication use (see Figure 2).

The defining characteristic of MEI models is all items measure *related *aspects of the same satisfaction-dissatisfaction construct (Convenience). As a result, all three of these TSQM items are essentially interchangeable with one another and item measurements are expected to be highly intercorrelated with one another. Moreover, when computing a scale score, the precision of the construct estimate is increased by averaging out random measurement error associated with any single item rating – theoretically, providing an error free estimate. Moreover, if any one of the item measurements provides a perfect construct estimate (i.e., contained no measurement error) there would be no additional benefit gained by including any of the remaining scale items.

### Single Effect Indicator models

Model A1 depicts a Single Effect Indicator (SEI) model being used to measure a latent construct. As mentioned, the SEI model is a special case of the MEI model, where a single item is assumed to be single best and least error prone measure of the latent construct. The addition of more items would not contribute significantly to the precision of the construct estimate. Some constructs may be better assessed using a SEI model and a single item indicator than others. Pain severity assessment is one such example, where experience tells us that it is difficult to design different measures of pain severity, especially ones that have uncorrelated errors.

The SEI model can be expressed in structural equation terms as in equation [2]:

*Y*_1 _= λ_I1_η_1 _+ ε_1 _    [2]

Where, *Y*_1 _is the single symptom or general measure, η_1 _is the underlying latent construct, λ_I1 _is the factor loading that is fixed at "1.0" and ε_1 _is the error term that is fixed at "0". As the equation implies, the item is assumed to precisely measure the construct. The sole use of a single item, however, provides no way to evaluate the relative impact of measurement error on either the reliability or precision of a construct estimate, and typically this sort of information is only available from previous validation studies.

**Figure 3:**
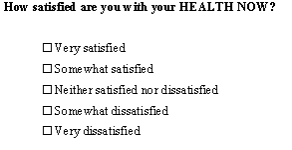
A general rating scale of 'Current Health' used in the Health Assessment Questionnaire.

Many PRO measures include a generally worded single item indicator measure instead of, or in addition to, using a multiple item scale. An example of a generally worded SEI indicator of current 'Health' is included in the Health Assessment Questionnaire (see Figure 3). Whether used knowingly or unknowingly, the use of general wording is one way to reduce the unexplained variance across heterogeneous samples, since ratings of more specifically worded items is influenced to a greater extent by individual differences and situational characteristics than ratings of generally worded content. Generally worded items allow respondents the freedom to interpret the meaning of a question and provide ratings based on their own unique experiences and life circumstances. This permits estimation of a general construct over very different observational contexts and respondent groups, since the much of the conditional variance remains essentially unaddressed or inferred [19]. We will return to this point when discussing the activities associated with item design and content validation using various measurement models in Part II of this commentary.

### Multiple Cause Indicator models

A very different family of models is used to estimate a construct when its' various indicators are not expected to be highly correlated, but instead ask about somewhat unique aspects of the construct. Application of a Multiple Cause Indicator (MCI) model is based on the premise that each observation uniquely contributes to the precision of the overall estimate of the latent trait or condition. The most distinctive aspect of this model is that items are not interchangeable or even necessarily similar to one another. This differs from the statistical relationship between items in a MEI model, where the latent construct itself is defined by the covariance of related observations across a group of respondents. The psychometrically astute reader may recognize the distinction between MEI and MCI measurement modes as similar to the statistical distinctions between factorial analytic and regression approaches when using respondent-based measures.

Model B in Figure 1 visually depicts an MCI model using structural equation notation. Compared with MEI and SEI models, the direction of the arrows is reversed and the model lacks error term for each of the observed indicators. A disturbance term (ς_1_) on the latent construct indicates the proportion of variance in the latent construct which is *not *accounted for by the weighted linear combination of the measured items. The MCI model is presented mathematically in Equation [3]:

ξ_1 _= λ_1i_x_1 _+ λ_12_x_2 _+ λ_13_x_3 _... λ_1n_x_n _+ ς_1 _    [3]

Where: ξ_1 _is the latent construct, x_i _represents the measured causal indicators, λ_1i _is the coefficient weight linking the causal indicators to the latent construct and ς_1 _is the disturbance in the latent construct described above.

Since the construct is not identified internally by the (error free) statistical intersection of observed ratings, as it is in the MEI model, the importance and weights associated of each indicator must be established against a criterion variable used as proxy for the latent construct. Such proxy criteria are typically considered 'gold standards' in the field and may include diagnostic clinical interviews, laboratory classification, or very well established self-report measures of the construct of interest.

The Disability Index of the Health Assessment Questionnaire [20] has the look of a scale based on a MCI model (see Table 1). First, the items are clearly distinct and not interchangeable indicators of the underlying construct. For example, being unable to tie one's shoe is not the same as being unable to stand up. The performance of these activities relies on a different set of motor skills associated with differences in dexterity, balance and strength. Second, given the differences between items, the score estimate of total disability is based on a cumulative index of a number of different types of physical skills that a patient has difficulty performing. Thus the combination of individual MCI items is thought to assess the summative effect of unique aspects of disability rather than assess the true score for a specific type of skill deficit.

**Table 1:** A Multiple Cause Indicator Measurement Model in the HAQ Disability Index

Can you:	Without ANY Difficulty	With SOME Difficulty	With MUCH Difficulty	Unable To Do
Dress, including tying shoelaces	□	□	□	□
Shampoo your hair	□	□	□	□
Stand up from a straight chair	□	□	□	□
Get in and out of bed	□	□	□	□
Cut your meat	□	□	□	□
Lift a full cup or glass to your mouth	□	□	□	□
Open a new milk carton	□	□	□	□
Walk outdoors on flat ground	□	□	□	□
Climb up five steps	□	□	□	□
Wash and dry your body	□	□	□	□
Take a tub bath	□	□	□	□
Get on and off the toilet	□	□	□	□
Reach and get down a five pound object	□	□	□	□
Bend down & pick up clothing from floor	□	□	□	□
Open car doors	□	□	□	□
Open jars which have been opened	□	□	□	□
Turn faucets on and off	□	□	□	□
Run errands and shop	□	□	□	□
Get in and out of a car	□	□	□	□
Do chores i.e. vacuuming or yard work	□	□	□	□

Symptom checklists and symptom severity measures are other examples of PROs which are often based on an MCI measurement model. Like the items used in the Disability Index, symptom severity items are often discrete due to differences across individuals in both the symptomatic expression of illness and the relative impact particular symptoms on overall ratings of symptom severity. As a result, one would expect such ratings to be more weakly correlated, exhibit more statistical independence, and have more skewed distributions (e.g., floor effects) than the interchangeable items used within an MEI scale. Moreover, the differential impact of certain types of symptoms on overall symptom severity may suggest that the item ratings need to be 'impact' weighted in order to provide the best estimate of the measurement construct.

### Single Cause Indicator models

Model B1 in Figure 1 illustrates the Single Cause Indicator (SCI) model, a less common variant of the MCI model, in which a single indicator is thought to be the single primary cause of a latent construct. The addition of other items which assess different causal determinants should not dramatically improve the predictive power of a measure appropriately based on a SCI model.

Equation [4] describes the SCI model in structural equation terms:

ξ_1 _= λ_11_x_1 _+ ς_1 _    [4]

Where, ξ_1 _is the latent construct, x_1 _is the single causal indicator, λ_11 _is the coefficient connecting the single indicator to the latent constructs, and ς_1 _is the variance not explained in the latent construct. Like the MCI model, the strength of the causal relationship between the item and construct is defined using a proxy measure of the latent construct. The weight estimate of the indicator is essentially the amount of variance in the latent construct that the indicator explains.

**Figure 4:**
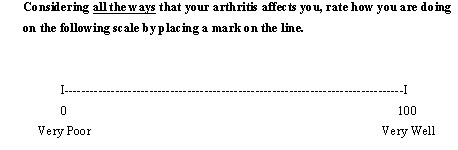
A 'Health Status' visual analog scale used in the Health Assessment Questionnaire.

A general SCI summary rating is sometimes used as a substitute for longer MCI measures. An example is a summary judgment scale used in the Health Assessment Questionnaire, the Health Status Visual Analog Scale (see Figure 4). This single item asks respondents to consider 'all the ways' the disease affects them and the single item presumably reflects the mental combination of a number of different (perceived) arthritic causes of their overall health status. As discussed earlier, details about causes that impact respondents' overall rating are unspecified and allowed to differ across individuals. As a result, such items tend to provide more normally distributed scores than content-specific MCI items which may be relevant to a proportion of respondents.

### Multiple Mixed Indicator models

One final family of measurement models is what Bollen and Lennox [21] refer to as the Multiple Mixed Indicator (MMI) model. As the name implies, such models contains a combination of items to measure the causes and effects of two or more latent constructs and are diagrammatically represented by combining two or more of the four basic measurement models. In some cases, these latent structures are hierarchical, for example, when a general construct is thought to be the effect of a series of more specifically defined latent constructs and content-specific items (see Figure 2) [22]. As will be discussed further in Part II, MMI models can be used to provide support for the structural validity of either MCI, SCI or SEI measures which require a criterion measure to estimate the latent construct.

Unfortunately, explicit MMI modeling does not often occur by design but usually occurs when scale constructors fail to distinguish between the different types of relationships between observed measures and latent constructs. Bollen and Lennox [21] utilized the Center For Epidemiologic Studies Depression Scale (CES-D) as such an example, in which they pointed out that the item that measures feelings of sadness or of being depressed appears to be the effects of depression; whereas items that assess loneliness may be caused by depression and items that measure perceptions of attractiveness may be reciprocally related to the depression. Such problems are very common and the astute reader may be able to identify a minor area of model mis-specification in Figure 2.

## Part II: Measurement models and different validation methods

Prior specification of the types of relationships between items, scales, and their underlying constructs provides a testable model for subsequent validation activities. The measurement model structures the PRO so that assessments closely mirror how the characteristics of interest are thought to be manifest in the target population(s), and so that the resulting construct estimates best suit the purpose(s) for which the measure is intended. Thus specification of the measurement model(s) to be applied to the item pool, ideally occurs before large-scale psychometric testing begins.

### Thematic relevance and item design

A first step in the design of most new measures is specification of a modifiable conceptual framework that both guides, and is refined by, qualitative inquiry within patient focus group sessions [23]. The degree of conceptual focus or structure may vary based on the measurement domain and purpose of measurement. The organization and direction of qualitative inquiry should itself be the subject of focus group review, as early content validation activities ought to lean towards an inductive expansion of knowledge, not a deductive confirmation of preconceptions and models. The resulting qualitative findings provide the thematic basis for item design, which eventually leads to the composition of a testable item pool. Importantly, qualitative findings and the elaborated conceptual framework also inform the selection of an appropriate measurement model for broader deductive validation activities.

The qualitative themes and resulting PRO item content to be used in MEI-based measures will typically be relevant to the majority of individuals in the focus groups and, by extension, the target assessment population. Moreover, MEI items will seem to be thematically clustered by concept and the items should appear to measure very similar aspects of the underlying concept. The thematic similarities between three or more items should be shown empirically to structurally define a MEI-based scale. When PRO item content covers a range of apparently unrelated themes and the importance of such content differs across individuals within the focus groups, a MCI model may be a better choice to guide measurement design. The objective of a MCI approach is to account for as many significant causes of a construct as possible, even if a particular cause is only important to a small subset of individuals [24]. Because of the central role of item relevance when selecting a measurement model, obtaining importance and/or frequency ratings from focus group participants on all draft items is an extremely informative, yet often neglected aspect of early PRO design activities.

### General versus specific item content

Schwartz and Rapkin [25] expand on the important distinction between items that are more generally worded, referring to broad concepts (i.e., high bandwidth items) and items that refer to detailed content associated with a concept (i.e., high fidelity items). More generally worded items seem relevant to a greater proportion of respondents than items that refer to specific content – thus resulting in general items eliciting higher importance ratings across the sample than more specifically worded items. As discussed earlier, generally worded, high bandwidth, items allow respondents' the flexibility to make ratings based on what they consider are related experiences and this set of experiences need not be the same for all individuals. When probed about specific reasons underlying respondents' rating of a general item, they may refer to different experiences based on the particulars of their condition and its' unique impact on various aspects of their lives. Because of the flexible ways generally worded items are interpreted, they tend to appear relevant or valid to most or all respondents [26], a characteristic of both specific and general items used in appropriately modeled MEI measures. This characteristic helps explain why high bandwidth items are found to work well across different patient populations and be good predictors of events which are influenced by individuals' evaluation of their unique health-related experiences [22,27,28].

A practical consideration when using generally worded measures is they do not provide much insight into the specific meaning of the construct nor the reasons underlying variations in score estimates. This uncertainty can lead to difficulties when interpreting score differences, particularly across groups with different health conditions. For example, patients suffering from osteoarthritis might be interpreting a general question about current health status very differently than those who suffer from migraine. Such 'referential uncertainty' may present a problem when a PRO will be used to justify specific claims about a product or service [23]. On the other hand, as the content of items is made more specific it is likely that the items will become less relevant to an increasing proportion of the sample. Symptom severity rating scales, for example, often contain a number of different symptoms of illness that not all respondents experience a problem with. Interested readers are referred to [25,26] for a more detailed discussion on the topic of addressing content generality and specificity of PRO items.

### Differing approaches to structural validation of PROs based on MCI and MEI models

Moderate to high correlations and high internal consistency estimates between items is a desirable psychometric characteristic, but only if a scale was constructed on the classical MEI model. Factorial and IRT analyses, both MEI validation methods, are based on the assumption that unidimensionality of items identify those best suited for construct estimation. When designing measures based on the alternate MCI model, however, high internal consistency estimates may actually indicate excessive co-linearity between item ratings and possible misspecification of the measurement model.

As a result, assessment of the internal consistency and factorial structure of true MCI measures may appear disappointingly weak, when in fact such statistical methods may not be appropriate ways to assess the ability of MCI items to estimate the construct of interest. The valid selection of MCI items ought to be based on assessment of the discriminative or predictive power of candidate items to independently explain significant variation in a dependent criterion measure. Such an approach is commonly used outside of the field of Outcomes Research to validate diagnostic classification and clinical assessment measures [29-31]. Other than psychometric tradition in Outcomes Research, there does not seem to be any good reason why such methods should not be adopted by those in the field of PRO instrument design.

There are numerous examples of validation activities where the distinctions between different validation methods associated with MCI and MEI models become blurred or are not considered. Piland and colleagues [32] for example, present evidence for construct validation of a symptom assessment measure using MEI validation methods. Thus, as might be expected, their final confirmatory structural equation model includes only items from the test pool that are the least skewed, indicative of their greater relevance to the overall sample. It is unclear if an MCI model were applied, whether the dropped items would have added either precision to estimates of patients' symptom distress or predictive power against a meaningful clinical criterion. It would be very helpful if procedural criteria were available to guide decisions about when a particular set of items are more appropriately described using a MEI or MCI model, and whether inclusion of both types of items provide a better estimate of the construct.

### Construct validation of single item indicators

When advocating the use of single item measures, validation activities need to provide evidence that the proposed item is the dominant cause or effect of the measurement construct and that the addition of other candidate items will not significantly improve measurement precision. The selection of a single cause or effect item occurs as a special case of an MCI or MEI model, and evidence to support the validity of a SEI or SCI model should arise out of psychometric evaluation of an MEI or MCI item pool.

The following results from MEI structural validation activities may suggest that it would be acceptable to use of a single item SEI scale: All major MEI candidates are very highly intercorrelated (>.90), items possess very high factor loadings (>.90), the item-scale correlations are very high (>.90) and the Cronbach's Alpha coefficient for the MEI scale is very high among candidate items (>.90). An SCI candidate, on the other hand, should be moderate to highly correlated (>.7) with a gold-standard criterion/proxy measure of the latent construct, and importantly, no other additional candidate MCI items should enter a regression or discriminant validation equations as significant predictors of the criterion measure. Given the rigor of these requirements, with a few notable exceptions (e.g., pain ratings), it is perhaps understandable why many single item measures tend to broadband measures of a general construct, since such items are largely unaffected by unexplained variance associated with individual and situational differences.

### Advantages of a mixed model approach to construct validation

Till now, for the sake of clarity, MCI and MEI models have been considered distinct ways in which to model and derive construct estimates. In practice, however, a constructs may best be estimated or validated using a combination of both approaches. For example, estimates of symptom severity may be best approximated by some combination of scores on a MEI scale composed of item shown to be relevant to all or most respondents, and a MCI scale containing items relevant to subgroups of individuals. There are a number of examples of PRO measures, most notably in the area of oncology, which do for this very reason, include both scales which are relevant to all respondents as well as scales composed of items that may only be important to subsets of respondents [33-35].

Due to the requirements of MCI models for an 'external' criteria against which to evaluate candidate items, a mixed hierarchical model has proved useful to assess the construct validity of such MCI PROs [36-38]. Such approaches use either a generally worded summary SCI/SEI item or a generally worded MEI scale as a broadband (dependent) criterion against which to identify the MCI items which independently contribute to the explained variance on the general construct. A general MEI measure should theoretically provide a construct estimate that approximates what one might expect from a well constructed (high fidelity) MCI measure of the same construct, and the fit index of such a mixed model should be quite high. If this is observed, a case can be made that the specific items represent the most important causal predictors of the general construct and therefore would comprise a structurally valid MCI scale. Additionally, the order of entry and slope coefficients associated with MCI items provide some sense of their relative importance as causes of the measurement construct.

Finally, there are numerous examples of MCI-based assessments within the clinical research literature that are not typically thought of as providing an estimate of a latent construct. Rather, these self-report or interview-administered assessments are used to make a medical prediction, judgment or decision [39]. Examples of such measures include screening tools and diagnostic measures, where the validity of the measure is assessed using (probabilistic) classification methods against a clinical standard, including demonstration of the positive/negative predictive value and sensitivity/specificity of resulting classifications. While differing from traditional trait theory in which measurement constructs are rarely estimated discretely, there are no good reasons why construct estimation cannot result in discrete classifications. Interestingly, the statistical approaches for validation of screening and diagnostic self-report assessments are wholly consistent with MCI validation methods presented in this article, since the validity of such measures is based on evidence from logistic regression, discriminant, and Receiver Operator analyses [39-41].

### Different scaling approaches

Typically, the constructs assessed by MEI measures are estimated using the mean or transformed mean of all items comprising a scale, thereby removing unexplained (error) variance associated with any single item. Weighting by factor loadings or item importance ratings has not been found to significantly improve the precision of construct estimation of MEI scales since items are highly correlated and already interchangeably important to all respondents [42,43]. Although score weights provide by IRT analyses has been shown to allow for more responsive coverage of the range of possible score estimates on the latent construct.

On the other hand, the responsiveness, predictive strength, and discriminative power (e.g., sensitivity and specificity) of MCI measures can be significantly weakened by using a mean score computations across items which are not of great concern to many respondents. This risk of weakened precision is heightened when insufficient attention has been paid to removal of non-significant MCI items during structural validation activities or when the chosen regression model has not been structurally validated in the sample being assessed [44].

In order to increase the precision of the measurement estimate, as well as its' predictive and discriminant power, MCI items are often 'relevance scored' using regression weights (a unit-weighted *sum *across all items). Within the clinical literature, other forms of relevance scoring are based on discrete or discontinuous algorithms which calculate an estimate for individuals only using those items for which they have reach a certain threshold. The use of threshold criteria with MCI items has been shown to increase the positive predictive value as well as sensitivity of their use as clinical screening and diagnostic tools [45,46].

### Summing up

Differences between cause and effect measurement models are not often brought into such close juxtaposition as in this commentary. Table 2 presents our view of the dimensions of measure which tend differ between these two types of measurement models. It is our hope that the materials presented here will spurn new ways of thinking about, and designing PROs with greater measurement precision.

**Table 2:** A continuum exists between MCI and MEI measurement models.

***Causal Indicator Model***	**Model Characteristic**	***Effect Indicator Model***
Item content assesses any cause of the measurement construct that is relevant to a (sub)group of respondents	**Content Relevance**	Item content assesses the effects of the measurement construct that is relevant to all or most respondents

Item content tends to be specific (high fidelity)	**Content Specificity**	Content may be either specific or general

Item ratings exhibit statistical independence and contribute unique predictive power	**Association between Item Ratings**	Item ratings are highly correlated, canceling random measurement error

Item ratings are skewed due to differential content relevance across respondents	**Item Score Distributions**	Item ratings are normally distributed due to common relevance of item content

Multivariate regression, cluster, and discriminant analyses against a criterion estimate (of the latent construct)	**Construct Validity Statistics**	Factor and IRT analyses of item covariance or response probability patterns

## Conclusion

In this paper we provide an overarching framework for recognizing the fundamentally different families of measurement models and the functional relationship between the measured items and the latent construct they presume to measure. The choice of measurement model can be made more explicit based on consideration of the purpose of measurement, definition of the latent construct, and the relevance of measured items to respondents within the target population. Such measurement choices influence content development, item scaling, scoring algorithms and construct validation activities. Moreover, appreciation of various forms of measurement models will likely lead to better conceptual integration of measurement approaches used in Outcomes Research with the approaches used in other fields of clinical and epidemiological research.

**Figure 5:**
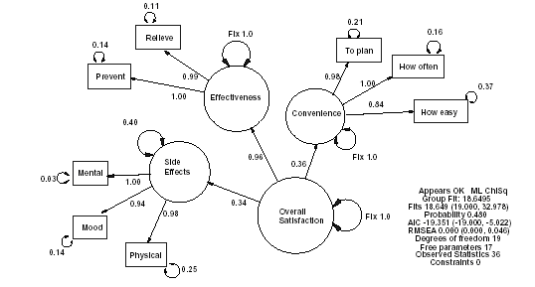
An example of a MMI model of Treatment Satisfaction.
